# The East Asian summer monsoon variability over the last 145 years inferred from the Shihua Cave record, North China

**DOI:** 10.1038/s41598-017-07251-3

**Published:** 2017-08-01

**Authors:** Xianglei Li, Hai Cheng, Liangcheng Tan, Fengmei Ban, Ashish Sinha, Wuhui Duan, Hanying Li, Haiwei Zhang, Youfeng Ning, Gayatri Kathayat, R. Lawrence Edwards

**Affiliations:** 1 Institute of Global Environmental Change, Xi’an Jiaotong University, Xi’an, 710049 China; 2 Department of Earth Sciences, University of Minnesota, Minneapolis, Minnesota 55455 USA; 3 State Key Laboratory of Loess and Quaternary Geology, Institute of Earth Environment, Chinese Academy of Sciences, Xi’an, 710061 China; 4 Faculty of Environmental Economics, Shanxi University of Finance & Economics, Taiyuan, 030006 China; 5 Department of Earth Sciences, California State University Dominguez Hills, Carson, 90747 USA; 6 Key Laboratory of Cenozoic Geology and Environment, Institute of Geology and Geophysics, Chinese Academy of Sciences, Beijing, 100029 China

## Abstract

The precipitation variability associated with the East Asian summer monsoon (EASM) has profound societal implications. Here, we use precisely dated and seasonally-resolved stalagmite oxygen isotope (δ^18^O) records from Shihua Cave, North China to reconstruct the EASM variability over the last 145 years. Our record shows a remarkable weakening of the EASM strength since the 1880s, which may be causally linked to the warming of the tropical Pacific and Indian Oceans. The δ^18^O record also exhibits a significant ~30-year periodicity, consistent with the instrumental, historical and proxy-based rainfall records from North China, plausibly driven by the Pacific Decadal Oscillation (PDO). Together, these observations imply that ~30-year periodicity is a persistent feature of the EASM, which remains significant with or without anthropogenic forcing. If indeed, the EASM rainfall in North China might decline significantly in the near future, which may affect millions of people in this region.

## Introduction

The East Asian summer monsoon (EASM) is a major source of moisture to eastern China, imposing critical influence on the lives of hundreds of millions of people in the region^1–3^. Over the past few decades, the EASM has exhibited a weakening trend^4^, marking a major climate shift in eastern China since the late 1970s^5–11^. This weakening trend also appears to have intensified after the early 1990s^5^. A precise characterization of EASM variations on decadal-multidecadal timescale is important for predicting the monsoon rainfall behavior in the near future. A number of mechanisms have been proposed to explain this declining trend in EASM rainfall as well at its decadal-multidecadal variability. These mechanisms include: (1) variations in snow cover over the Tibetan Plateau^6^, (2) oscillations in atmospheric circulation from high latitude^11, 12^, (3) oscillations in atmospheric circulation from lower latitude^9^, (4) variations in tropical Indian and/or Pacific sea surface temperature (SST)^6, 8, 13^, (5) the PDO^12, 14–16^ and (6) aerosol forcings^17^. However, the dynamical mechanisms that drive EASM variability on these timescales remain unclear^10^ due, in part, to the brevity of instrument records.

Tree-ring records and historical accounts have previously been used to reconstruct rainfall variations over monsoonal China^18–22^. For instance, a drought/flood index derived from a large set of Chinese historical records^20^ was used to reconstruct the EASM precipitation variations and its distinctive regional patterns over the last 530 years^19, 21, 23^. Kang and Yang^19^ reconstructed the variations in the annual rainfall between 1470–2000 AD by combining tree-ring records with historical documents from the fringe areas of EASM, where precipitation variance and ecological system, are supposedly more sensitive to variations in the EASM strength^7, 14, 18, 21, 24^. However, tree-ring and historical records are, in principle, proxies that mainly indicate local climate variations rather than large-scale supra-regional changes in EASM.

Previous studies have demonstrated that stalagmite δ^18^O based reconstructions of the EASM are comparable with instrumental and historical records^25–30^. The stalagmite δ^18^O records have been shown to represent large-scale and spatially-integrated monsoon rainfall between the tropical monsoon moisture sources and the cave sites^31^. Currently, the improvements in sampling techniques make it possible to obtain seasonal or even weekly-resolved stable isotope records from stalagmites with moderately fast growth rates^32, 33^. In this study, we present a first seasonally-resolved stalagmite δ^18^O record from Shihua Cave, Beijing, North China over the last 145 years. We demonstrate that the δ^18^O record mainly reflects variation in the EASM intensity and/or regional precipitation. Our record shows a centennial-scale weakening trend in the EASM since the 1880s, which is superimposed by multidecadal oscillations that are possibly linked to the PDO.

## Cave location, climate, and sample

**Figure 1:**
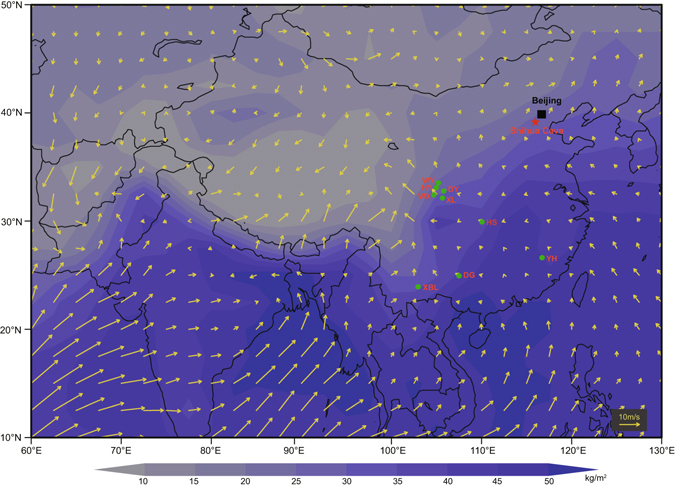
Precipitable water content with surface wind field and locations of Shihua and other caves. Rainy season (June–September) precipitable water content (kg/m^2^) for the entire atmospheric column with surface wind field averaged during 1987–2016 AD based on the NCEP/NCAR reanalysis datasets^34^. The shading shows absolute value of precipitable water content and the arrows indicate the surface wind field. The location of Shihua Cave is represented by red star (this study), and the others by green circles: WY (Wuya Cave)^25^, HY (Huangye Cave)^28^, WX (Wanxiang Cave)^29^, DY (Dayu Cave)^26^, XL (Xianglong Cave)^32^, HS (Heshang Cave)^35^, YH (Yuhua Cave)^36^, DG (Dongge Cave)^37^, XBL (Xiaobailong Cave)^27^. The map is created using Open Grid Analysis and Display System (OpenGrADS, the software (Version 2.0.2.oga.2) is available at https://sourceforge.net/projects/opengrads/).

Shihua Cave (115°56′E, 39°47′N, 251m above sea level at the entrance) is located in Fangshan County, ~50 km southwest of Beijing, North China (Fig. 1). The region is characterized by cold/dry winter and warm/wet summer because of its location at the fringe of the EASM influenced-region. The mean annual temperature and precipitation between 1980 and 2015 AD are 12.9 °C, and ~536 mm, respectively. More than 70% of the total annual precipitation falls during the summer monsoon season (June to September). The host bedrock of the cave is the Middle Ordovician limestone (the Maojiagou Formation) with some interlayers of dolomite. The thickness of the bedrock above the cave varies from ~30 to 130 m. The cave has multi-levels and many branches. Hitherto, seven levels have been explored. An actively-growing stalagmite XMG-1 with a diameter of 40 mm and height of 36 mm, was collected from the third level of the cave in October 2015. Prior to the collection, calcite deposition over a glass plate, placed directly above the XMG-1, was observed over a half-year monitoring period, confirming that the stalagmite was actively growing before the collection date.

## Results

### Chronology

We used ^230^Th dating in combination with annual layer count to establish the age model of XMG-1, and the result was further verified by annual δ^13^C cycle counting. Five ^230^Th dates in stratigraphic order provide the first-order constraint on the XMG-1 chronology. A total of 143 annual layers were counted with an accumulated error of 7 years at the bottom, consistent within the margin of error of the ^230^Th dates (see Methods, Supplementary Fig. S3). In addition, the annual layer count using confocal microscopy^38^ (see Methods) corresponds well with the δ^13^C cycle-based count (Supplementary Fig. S2). The annual δ^13^C cycles presumably result from humid and warm conditions in summer, which are associated with more vegetation and microbial activity, and consequently more organic carbon in percolating water and thus lighter δ^13^C values. In contrast, the process in winter is expected to be opposite because cold and dry conditions would result in higher δ^13^C values^39–41^. In addition, the dry winter conditions may also lead to a longer residence time of seepage water and may enhance prior calcite precipitation (PCP), leading to higher δ^13^C values in the winter portion of the annual layer^39, 41^. The total counts of δ^13^C cycles range from 131 to 165 over the entire sample (see Methods), consistent with ^230^Th-dating/layer-counting derived age model within uncertainty. The broad agreement among the approaches using different dating methods demonstrates that our age model is robust. The progressively accumulated error of 7 years at the bottom is sufficiently small for addressing ~100-year trend and multidecadal variations that are the primary focus of this study.

### Cave δ^18^O proxy

**Figure 2:**
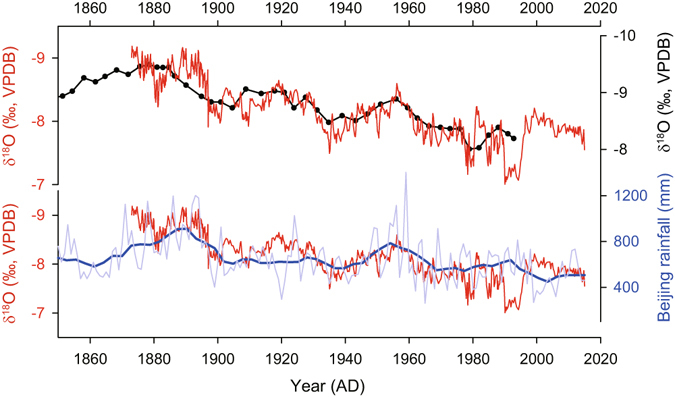
Comparison between the Shihua record with other records from Beijing. (**a**) Comparison between the Shihua XMG-1(red) and S312^43^ (black dotted line) δ^18^O records. (**b**) Comparison between the Shihua XMG-1 record (red) and annual precipitation record in Beijing (blue)^46^. The thick line is the 5-year running mean curve.

The use of stalagmite δ^18^O as a valid proxy of the δ^18^O of meteoric precipitation (δ^18^O_p_) requires establishing that the stalagmite in question was deposited under the oxygen isotope equilibrium conditions. We applied the “Hendy Test”^42^ on five growth layers of XMG-1, which indicates that the δ^18^O values are virtually constant along a single layer (Supplementary Fig. S5). Additionally, a broad resemblance between our XMG-1 δ^18^O record and a previously published δ^18^O record (S312)^43^ from the same cave passes the so-called “Replication Test”^44^, confirming that kinetic fractionation is negligible^44, 45^ (Fig. 2). Consequently, we interpret the XMG-1 δ^18^O variations as a proxy for the δ^18^O_p_ changes.

Although stalagmite δ^18^O values represent regional δ^18^O_p_, the climatic interpretation of δ^18^O_p_ remains a hotly debated issue, especially on centennial to decadal scales^32, 35, 37, 43, 47^. This is because the δ^18^O_p_ can be influenced by a number of factors such as air temperature, precipitation amount, moisture source and fractionation during transport processes^48, 49^. Cheng *et al*.^31^ recently suggested that lower δ^18^O values in stalagmites from the EASM region implies, to a first order approximation, spatially-integrated monsoon rainfall between the tropical monsoon moisture sources and the cave site. In the ‘downstream’ or fringe areas of the EASM, lower δ^18^O values may also indicate higher summer monsoon rainfall on varying timescales^24, 25, 28, 29, 33^. This interpretation is consistent with the observed inverse relationship between our Shihua δ^18^O and instrumental rainfall records in Beijing on the centennial and multidecadal timescales (Fig. 2, r = −0.68 during 1873–2015 AD interval after applying a 7-year moving average, with 95% confidence interval (CI) [−0.63 −0.73]). Modern observations indeed support the idea that stronger EASM intensity could bring more summer rainfall with lighter δ^18^O values into North China^7, 14, 15^. However, the relationship between stalagmite δ^18^O and local precipitation is more complex on annual to interannual timescales, which may be caused by a ‘smoothing effect’ from water reservoir or ‘residual water’ effect in the vadose zone^32, 47^. In this contribution, we use the terms ‘strong monsoon’ and ‘weak monsoon’ to refer to low and high stalagmite δ^18^O values, respectively, consistent with the previous studies^24, 30, 31^.

### Cave δ^13^C proxy

The factors producing variation in stalagmite δ^13^C values are more complicated. These include: (1) production of biogenic CO_2_ by plant and soil processes, (2) ratio of C3 over C4 plants, (3) soil-water residence time, (4) CO_2_ degassing in the vadose zone, (5) contribution of bedrock δ^13^C and (6) isotopic composition of atmospheric CO_2_^26, 39, 41^. As mentioned above, wetter conditions favor relatively lower δ^13^C values. In addition, a wet climate is favorable of vegetation growth, which might lead to reduced δ^13^C values on decadal to multidecadal scales^26, 39^. Similar variations between our δ^13^C and δ^18^O records on annual to decadal scales agree with this interpretation. The correlation coefficient (r) for the detrended δ^13^C (Δδ^13^C) and δ^18^O (Δδ^18^O) records is 0.43 (*p* < 0.01, n = 686).

## Discussion

**Figure 3:**
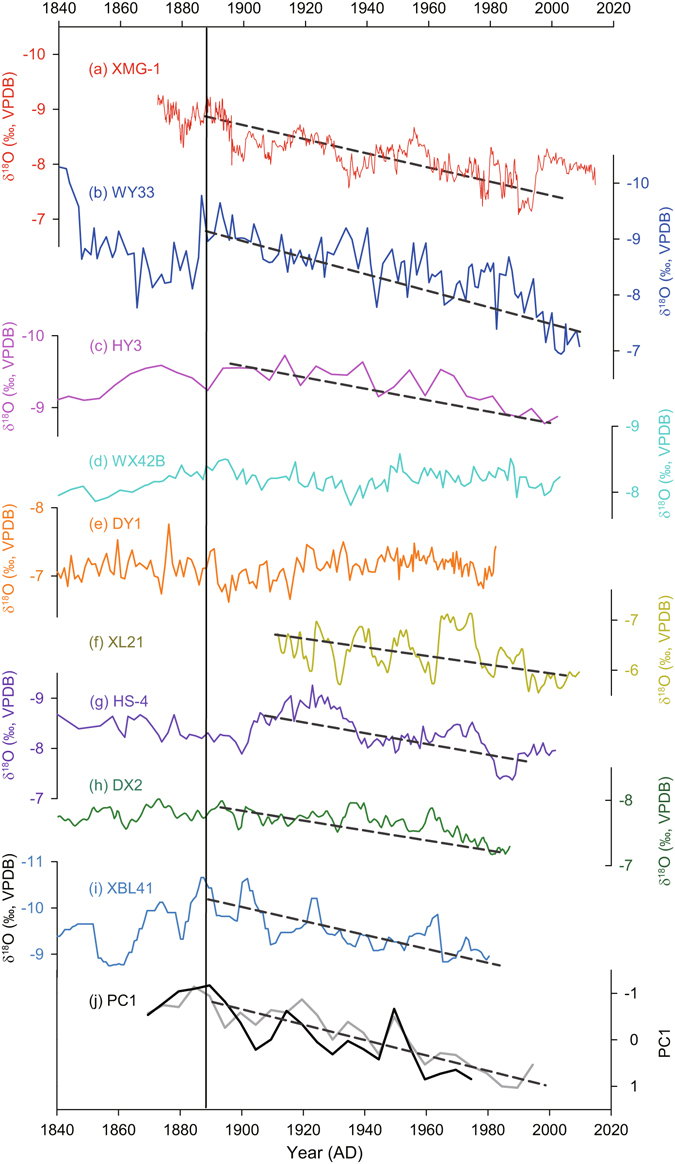
Comparison between Chinese stalagmite δ^18^O records since 1840 AD. (**a**) to (**i**) are Shihua Cave (red, this study), Wuya Cave (blue)^25^, Huangye Cave (pink)^28^, Wanxiang Cave (Cyan)^29^, Dayu Cave (orange)^26^, Xianglong Cave (dark yellow)^32^, Heshang Cave (purple)^35^, Dongge Cave (green)^37^, and Xiaobailong Cave (sky blue)^27^ δ^18^O records, respectively. (**j**) shows two PC1 components (black, integrated from (**a**)–(**e**) and (**g**)–(**i**) records during 1875–1980 AD; gray, integrated from (**a**)–(**d**) and (**g**) records during 1875–2000 AD). The vertical line indicates the approximate beginning timing of increasing trend in these records identified by PC1 components (accounting for ~40% of the total variance), and the dark gray dashed lines depict the increasing trend in each record, except for WX42B and DY3 records which did not show a statistically significant trend since the 1880s.

The most prominent feature of our δ^18^O record is a centennial-scale increasing trend since the middle 1880s, indicating a progressive weakening of the EASM intensity (Fig. 3). This trend is also observed in many other stalagmite records from different locations in monsoonal China, including the southwestern China^27, 37^, southeastern China^36^, central China^32, 35^, the eastern part of northwestern China^25, 28^, and North China^43^. However, the Wanxiang and Dayu Cave records, located at a farther inland location in the eastern part of northwestern China do not appear to show a clear trend during this time period (Figs 1 and 3)—the reason of which deserves further investigation. The observed declining trend in EASM intensity as inferred from the aforementioned cave sites (Fig. 3), is well in line with a recently reconstructed rainfall record from the northern margin of the EASM, which also displays a declining trend since the 1880s^19^. In addition, it appears that the Indian summer monsoon (ISM) is also undergoing a similar weakening trend^50, 51^, suggesting that both subsystems of the Asian monsoon may share a common forcing, which is responsible for this centennial-scale weakening trend. We emphasize that the stalagmite δ^18^O records, as interpreted above, indicate changes in the overall EASM intensity or a first order change in spatially-integrated rainfall between moisture sources and cave site^31^. In other words, the weakening EASM trend inferred from the stalagmite δ^18^O records may just correspond to decreasing rainfall in certain regions, such as North China, but not necessarily in South China, because there is a ‘dipole mode’ of summer rainfall between North and South China as demonstrated by both instrumental and reconstructed precipitation records^7, 15, 30^.

**Figure 4:**
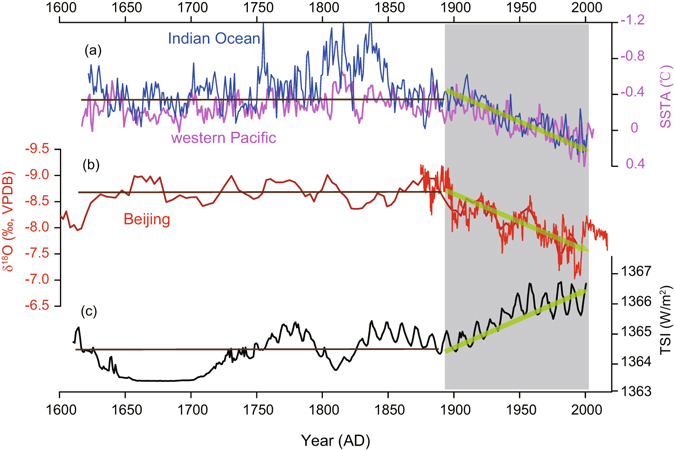
Comparison of climate records over the last 400 years. (**a**) Coral-based Sea Surface Temperature Anomaly (SSTA) in tropical Indian (blue) and western Pacific (pink) Oceans^55^. (**b**) The Shihua δ^18^O records (red, this study; dark red, S312)^43^. (**c**) Total solar irradiance (TSI, black)^56^. Arrows indicate the long-term variances and the dark gray rectangle shows the last century during which each record exhibits its distinct trend. Brown lines roughly depict the mean long-term variances before the 1890s.

The underlying causes of the EASM weakening trend remain unclear. Many studies have linked the weakening trend to the long-term warming of the tropical Pacific and/or Indian Oceans^6, 8, 13^. Based on instrumental data, Ding *et al*.^6^ illustrated a combined effect from the warming of the tropical Pacific SST and the increasing of winter and spring snow cover in the Tibetan Plateau on the weakening of the Asian summer monsoon since the second half of the 20^th^ century. Tan^47^ attributed the 20^th^-century increase in stalagmite δ^18^O values in eastern China to the decrease of the temperature gradient from the tropical Pacific, and thus to the long-term ENSO-like state. This trend is also considered to be a manifestation of the EASM multidecadal to centennial oscillations^10, 22^. Abnormal changes in the tropical Indian SST might also impose a significant impact on variations of the EASM as the cross-equatorial southwesterly is one of the main components of the EASM system^1, 2^. Our Shihua Cave record supports the idea that links the EASM weakening to the warming of the tropical SST over the last 400 years (Fig. 4). Notably, variations in solar irradiance in the last 100 years also display an increasing trend similar to the changes in the tropical SST anomalies (Fig. 4). Although the latter could explain the increase of the SST in tropical Indian and/or Pacific Oceans during the 20^th^ century, it is difficult to explain the observed EASM weakening prior to the 20^th^ century. This is because the increased solar irradiance would enhance the land-sea temperature gradient due to their differential thermal inertia, and in turn, strengthen rather than weaken the EASM^52, 53^. A recent study from the ISM domain^50^ showed that the strong positive coupling between Northern Hemispheric temperature anomaly and the ISM over the last 2000 years was broken during the last century, shedding new light on the paradox mentioned above. We thus propose that the EASM may also have decoupled with land temperature, which was presumably raised, in part, due to increase in solar irradiance over the last 100 years. This observation points to a possible role of the anthropogenic forcing in causing the weakening of the EASM, although it remains a subject of dispute^11, 54^. Regardless of the mechanisms, both the coupling between the EASM and the SST^6, 8, 13^ or solar irradiance/land-temperature^29, 52^ and the decoupling of the EASM from the solar irradiance/land-temperature would provide important constraints for understanding the EASM variability. Further theoretical and empirical studies are necessitated to explore the mechanisms behind the EASM weakening trend.

**Figure 5:**
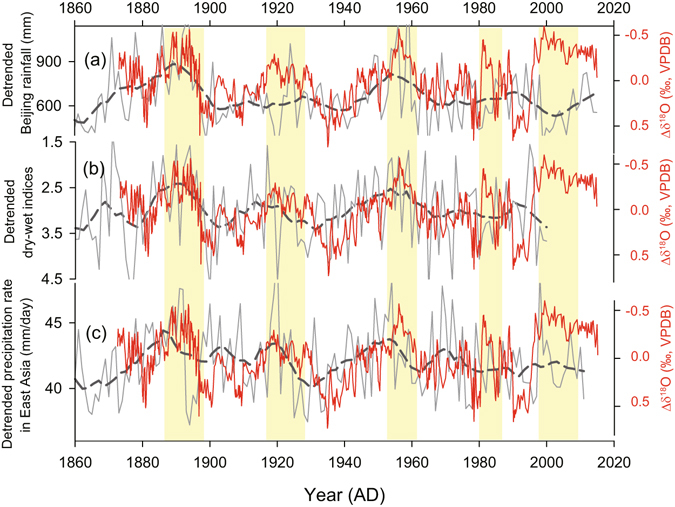
Comparison between the detrended Shihua δ^18^O record (red) and detrended precipitation dataset from different regions (gray curves). (**a**) Annual rainfall in Beijing^46^. (**b**) Dry-wet indices (DWI) in North China (37.5–42.5°N/112.5–117.5°E)^59^. (**c**) Annual precipitation rate in East Asia (20–45°N/110–140°E)^60^. The thin lines and bold dashed lines indicate annual and 7-year running mean curves, respectively. The yellow bars highlight the periods of lighter δ^18^O excursions.

**Figure 6:**
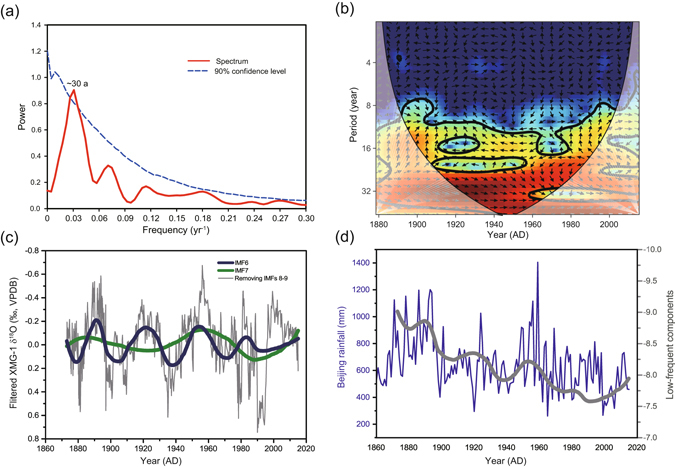
Time series analyses of the Shihua δ^18^O record and the PDO index. (**a**) Spectral analysis of XMG-1 δ^18^O record (red line) shows a period of ~30 years (labeled). The dashed line refers to the 90% confidence level. (**b**) The cross-wavelet transform of the PDO index and detrended XMG-1 δ^18^O time series (7-year moving average, 1873–2015 AD). The 5% significance level against red noise is shown as a thick contour. The relative phase relationship is shown as arrows (with in-phase pointing right and anti-phase pointing left)^61^. (**c**) Filtered XMG-1 δ^18^O record after subtracting the IMFs 8 and 9 from the original sequence (gray) (see Methods) superimposed by the IMF 6 (thick blue curve) and IMF 7 (thick olive curve), reflecting two modes of multidecadal oscillations (~30 and 50–70 years), respectively. (**d**) Beijing annual rainfall amount (blue) and the low frequcy variations in XMG-1 record represented by the IMFs 6–9 components (thick gray curve).

The long-term trend in XMG-1 δ^18^O record is superimposed by distinct decadal-multidecadal variability. In order to characterize the multidecadal variability, we removed the long-term trend in our δ^18^O record to obtain a detrended record (Δδ^18^O) (Fig. 5). Five strong EASM intervals are inferred from the negative Δδ^18^O excursions around the mid-1880s to early 1890s, 1920s, ~1950s, 1980s, and 2000s, respectively. In contrast, four weak monsoon intervals are found around the late 1890s to 1900s, 1930s to early 1940s, mid-1960s to 1970s, and 1990s, respectively (Fig. 5). Spectral analysis of the detrended record shows a statistically significant cycle of ~30-year (38–28 years, 90% confidence level) in our 145-year record (Fig. 6a and b). Our new record confirms a previous result from stalagmite S312 δ^18^O record^43^ from the same cave which also showed a similar periodicity. On the other hand, Tan *et al*.^25^ reported a significant quasi-50-year periodicity based on stalagmite δ^18^O records over the last 370 years from Wuya Cave, located farther inland in the eastern part of northwestern China. The apparent difference in the multidecadal-scale rhythm between the two records might be related to the dissimilarity in resolution and temporal range, resulting in a possible increase in higher frequency power over the lower frequency components in our record. In fact, another frequency component of multidecadal oscillation with a cycle of 50–70 years, also appears to be present in our Shihua record (Fig. 6c). Moreover, the ~30-year cycle has also been reported in the precipitation record in Beijing^57^ as well as from eastern China^7, 58^. Therefore, it appears that the ~30-year periodicity is a persistent feature of the EASM regardless of the apparent progressive increase of the anthropogenic forcing during the last century.

The multidecadal variability in summer monsoon precipitation integrated from East Asia (20–45°N/110–140°E)—covering eastern China, Korea, Japan and the adjacent marginal seas, also shows a striking similarity with our Shihua δ^18^O record (Fig. 5c, r = −0.4 after applying a 7-year moving average during 1873–2011 AD with 95% CI [−0.35 −0.45]). This observation is consistent with our interpretation of the Shihua δ^18^O record as a proxy of the overall monsoon strength indicating spatially-integrated monsoon rainfall between the tropical monsoon moisture sources and cave site^31^. According to the EASM dynamics, stronger EASM would transport more remote moisture to North China via stronger southerly wind flow and/or a longer summer monsoon season and *vice versa* ^7, 15, 21, 30, 47^. Both of these processes will, in turn, result in lighter δ^18^O of precipitation^48^. As such, one would expect that the Shihua δ^18^O record should have an inverse relationship with rainfall in North China. Indeed, the Shihua δ^18^O record shows a remarkable similarity with the dry-wet indices in North China (Fig. 5b, r = 0.5 after removing linear trend and applying a 7-year moving average during 1873–2000 AD with 95% CI [0.48 0.58]), as well as with Beijing annual rainfall (which mainly occurs during summertime) on multidecadal timescale (Fig. 5a). These observations further reinforce our interpretation of cave δ^18^O as a signal of overall monsoon intensity and/or North China rainfall.

Close similarities are also evident between the Shihua δ^18^O record and the PDO index on decadal to multidecadal scales with the strong EASM corresponding to cold (or negative) phase of the PDO and *vice versa* (Supplementary Fig. S6). The PDO is a manifestation of large-scale changes of the northern Pacific temperature pattern^62, 63^, which affects the climate in Asian-Pacific regions^23, 64^. Many studies have linked the PDO variance to decadal-multidecadal changes in rainfall or drought/flood conditions in North China^14, 16, 21^, the EASM intensity^14, 15^, and precipitation variations in eastern China^23, 58^. Model simulations also support the modulation of the PDO on summer monsoon precipitation in eastern China^65^. Earlier, Wang and Zhao^58^ have suggested that multidecadal variations in summer rainfall over eastern China are possibly subject to the air-sea interaction in the central tropical Pacific, which is considered as the equatorial lobe of the PDO^62, 63^. Subsequently, more studies have highlighted the relationship between the phase shift of the PDO and climate variability in EASM regions^12, 14, 15^. On the basis of statistical analyses, Zhu and Yang^14^ revealed a dynamic link of the PDO to the EASM or North China precipitation, which can be described as follows. When the PDO is in its warm mode, i.e., negative SST anomaly in the North Pacific and a positive anomaly in the central to eastern tropical Pacific, the negative sea level pressure anomalies during summer becomes much weaker in North Pacific, while positive anomalies strengthen over East Asia. Consequently, the Western Pacific Subtropical High shifts southward and the equatorial trade wind weakens, resulting in reduced EASM intensity and North China rainfall. Recent research from Qian and Zhou^16^ lent support to above interpretations. For instance, an abnormal heavy rainstorm occurred in Beijing on 21st July 2012, when the PDO was under strong cold phase (the PDO index was −2.34, significantly lower than that the long-term average of −0.34 from 1854 to 2015 AD).

Our Δδ^18^O record has a positive correlation with the PDO index on a decadal scale (r = 0.55 after applying a 7-year moving average during 1873–2015 AD, with 95% CI [0.47 0.61]) (Supplementary Fig. S6). This further supports the strong linkage of the PDO to overall rainfall variability in North China and/or eastern China (Fig. 5). In addition, we find the significant periodicity of ~30 years in our Shihua Δδ^18^O record, which is also a dominant periodicity of the PDO between 1873 and 2016 AD (Fig. 6b). Provided the current anthropogenic forcing have not change the EASM or North China rainfall natural periodicities, our record would therefore suggests a significant decline in the EASM and/or North China rainfall in about a decade, since the current mode of the relatively strong EASM and North China rainfall have lasted for ~15–20 years and thus is closer to the end according to the ~30-year periodicity (Fig. 6d). This is an important projection for millions of people living in North China with water availability in the region becoming crucial today and in the future.

## Conclusion

Based on the high-resolution and precise-chronology controlled Shihua δ^18^O record from North China, we reconstructed the variance in the overall EASM intensity and/or monsoon precipitation over the last 145 years. Our record shows a prominent decrease trend in the EASM since the 1880s, which coincides with a persistent warming in the Pacific and Indian Oceans. Accordingly, the amount of precipitation in North China is gradually decreasing over the same time period. Our record demonstrates that the EASM possesses a persistent multidecadal variability with a predominant periodicity of ~30 years, similar to the PDO, suggesting that the PDO may dominate the EASM variations on multidecadal timescales. Provided the ~30-year periodicity continues, our record suggests a possible drier climate that might occur in North China in the near future.

## Methods

### ^230^Th dating

The XMG-1 stalagmite was cut with wire line along its growth axis. Five subsamples for ^230^Th dating were drilled along the growth axis by using a hand-held carbide dental drill with 0.5 mm drilling bit. The ^230^Th dating was conducted in the Isotope Laboratory of Xi’an Jiaotong University, China, using a recently improved technique^66^. The procedure of chemical separation of Th and U is similar to that described in Edwards *et al*.^67^. Th and U isotopes were measured by a multi-collector inductively coupled plasma mass spectrometer (MC-ICP-MS, Neptune-Plus). The detailed instrumentation and technique are described in Cheng *et al*.^66, 68^.

### Stable isotope measurements

A total of 686 subsamples for stable carbon and oxygen isotope analyses were collected by using a micromill device at an interval of 0.05 mm. The stable isotope analyses were conducted in the Isotope Laboratory of Xi’an Jiaotong University, on a Finnigan-MAT 253 mass spectrometer connected with a Kiel Carbonate Device IV. Oxygen and carbon isotope ratios are reported in δ notation, δ^18^O and δ^13^C (‰), respectively, relative to the Vienna Pee Dee Belemnite (VPDB) standard. The international standard TTB1 were added to the analysis every 10 to 20 samples to check the reproducibility of results. Results show the typical analytical errors (1σ) for δ^18^O and δ^13^C are 0.06 and 0.03‰, respectively.

### Layer counting and δ^13^C cycles counting

We separate the XMG-1 sample into two sections to count the annual layers: (1) the top 4mm section (section A) and (2) the rest 31mm section (section B), due to their different surface features. In the section A, there are visible laminae with alternative transparent and white opaque layers (Supplementary Figure S1). A thin section was made to count the layers under a BX06 Olympus polarization microscope (4 × objective) using transmission light at the Key Laboratory of Cenozoic Geology and Environment, Institute of Geology and Geophysics, Chinese Academy of Sciences, China. We merged the photos together and counted the layers in multiple lines. A total of 25 transparent layers were counted. Second, the XMG-1 sample was imaged by a Nikon A1 Multiphoton confocal laser fluorescent microscope (CLFM) at the Lab of Bio-fabrication, School of Mechanical Engineering, Xi’an Jiaotong University, China. Fluorescence was stimulated with a 40 mW laser (488 nm wavelength), and a filter isolated the emitted wavelengths 515/30 nm — the green portion of the visible spectrum^38^. A series of overlapping images were obtained in order to generate a stitched map of the entire sample surface (10 × objective). Two orthogonal surfaces were scanned to allow cross-checking fluorescence layers. On the basis of the CLFM images, we identified 118 ± 7 fluorescence layers in the section B (Supplementary Figure S2). Furthermore, it is observed that the fluorescence layers correspond to the transparent laminae at the section A. Combining results from both sections A and B, we obtained 143 ± 7 annual layers for the whole section. The 7-year error is a progressively cumulative error at the bottom of the sample. On the other hand, it is also evident that the δ^13^C minima are concurrent with fluorescence layers very well (Supplementary Figure S2). To magnify the δ^13^C high-frequency signal, 4-point smoothing for the δ^13^C record was conducted and then subtracted from the original data to obtain the residual (Δδ^13^C) record. The Δδ^13^C record varies with amplitudes from 0.1 to 0.2‰—larger than the analytical error for δ^13^C (typically 0.03‰, 1σ). We then counted Δδ^13^C minima to obtain the total annual cycles. Using the Peak Analyzer (2 local points chosen for the Local Maximum Method) (OriginPro 2016 software, http://www.originlab.com), together with manual check, we obtain the counts of Δδ^13^C minima ranging from 131 (detected by software automatically) to maximally 165 years (from tentatively counting of all visible small Δδ^13^C peaks). The counting result of Δδ^13^C cycles is broadly in concert with that from annual layer counting, although the uncertainty is larger mainly due to the difficulty in assessing the small Δδ^13^C peaks.

### Data sources

The monthly precipitable water content for the entire atmospheric column and wind field at surface or near the surface (0.995 sigma level) (2.5° × 2.5°, from 1948 AD to the present) are from NCEP/NCAR Reanalysis Derived data provided by the NOAA/OAR/ESRL PSD, Boulder, Colorado, USA, from their website at http://www.esrl.noaa.gov/psd/. The monthly precipitation data (2.0° × 2.0°, 1851 to 2011 AD) in the East Asia region (20–45°N/110–140°E) from NOAA Twentieth Century Reanalysis Project version 2c dataset^60^ are integrated and downloaded from KNMI Climate Explorer (https://climexp.knmi.nl/selectfield_rea.cgi?id=someone@somewhere). The PDO index from the NOAA National Centers for Environmental Information (NCEI) is based on NOAA’s extended reconstruction of SSTs (ERSST Version 4)^69^ and constructed by regressing the ERSST anomalies against the Mantua PDO index^63^ for their overlap period (available at http://www.ncdc.noaa.gov/teleconnections/pdo/).

### Timeseries analyses

Confidence intervals of correlation coefficients were determined using the pairwise moving-block bootstrap method to preserve the serial dependence of time series and interval calibration to increase the accuracy^70^. Spectral analyses were computed using REDFIT software^71^ on the PAST platform^72^. The 90% confidence level is shown in Fig. 6. Principal Component Analysis (PCA) is used for capturing the common temporal-spatial pattern in diverse stalagmite records in eastern China. For analyzing, the stalagmite δ^18^O records with different duration and resolution were linearly interpolated into a 5-year resolution from 1875 to 2000 AD, and then calculated for PCA on the PAST platform^72^ in two strategies: (1) containing all the records between 1875 and 1980 AD except the XL21 record due to its short growing period; (2) including XMG-1, WY33, HY3 WX42B and HS-4 records between 1875 and 2000 AD (Fig. 3). We also linearly detrended all the data used in the PAST software^72^. The Ensemble Empirical Mode Decomposition (EEMD) analysis is a noise-assisted data analysis method that can objectively avoid the signal intermittency, and thus suitable for extracting signals from data generated in noisy nonlinear and nonstationary processes^73^. We did this analysis on the MATLAB workspace (R2015b version, http://www.mathworks.com) with the MATLAB code for EEMD available at http://rcada.ncu.edu.tw/research1_clip_program.htm. The EEMD method decomposes XMG-1δ^18^O record into nine Intrinsic Mode Functions (IMFs, we set the ratio of the standard deviation of the added noise and that of XMG-1 record as 0.2 and ensemble number for the EEMD as 300), among which IMF1 to IMF9 components have independent and meaningful frequencies varying from high to low, respectively (Fig. 6c).

### Data Availability

The data generated during the current study is available in the published article (and its Supplementary Information file).

## Electronic supplementary material

Supplementary information
