# Supplementary material for: The East Asian summer monsoon variability over the last 145 years inferred from the Shihua Cave record, North China
Source: Sci Rep. 2017 Aug 1;7:7078. doi: 10.1038/s41598-017-07251-3 (PMC5539322; doi:10.1038/s41598-017-07251-3)
Supplement: Supplementary file 1 — Supplementary information [file 41598_2017_7251_MOESM1_ESM.pdf]

Supplementary information for

The East Asian summer monsoon variability over the last 145 years inferred from Shihua cave record, North China

Xianglei Li[1], Hai Cheng[1,2,*], Liangcheng Tan[1,3], Fengmei Ban[4], Ashish Sinha[5], Wuhui Duan[6], Hanying Li[1], Haiwei Zhang[1], Youfeng Ning[1], Gayatri Kathayat[1], R. Lawrence Edwards[2]

[1]Institute of Global Environmental Change, Xi'an Jiaotong University, Xi'an, 710049, China

[2]Department of Earth Sciences, University of Minnesota, Minneapolis, Minnesota, 55455, USA

[3]State Key Laboratory of Loess and Quaternary Geology, Institute of Earth Environment, Chinese Academy of Sciences, Xi'an, 710061, China

[4]Faculty of Environmental Economics, Shanxi University of Finance & Economics, Taiyuan, 030006, China

[5]Department of Earth Sciences, California State University Dominguez Hills, Carson, 90747, USA

[6]Key Laboratory of Cenozoic Geology and Environment, Institute of Geology and Geophysics, Chinese Academy of Sciences, Beijing, 100029, China

Corresponding: cheng021@xjtu.edu.cn

Supplementary Figures

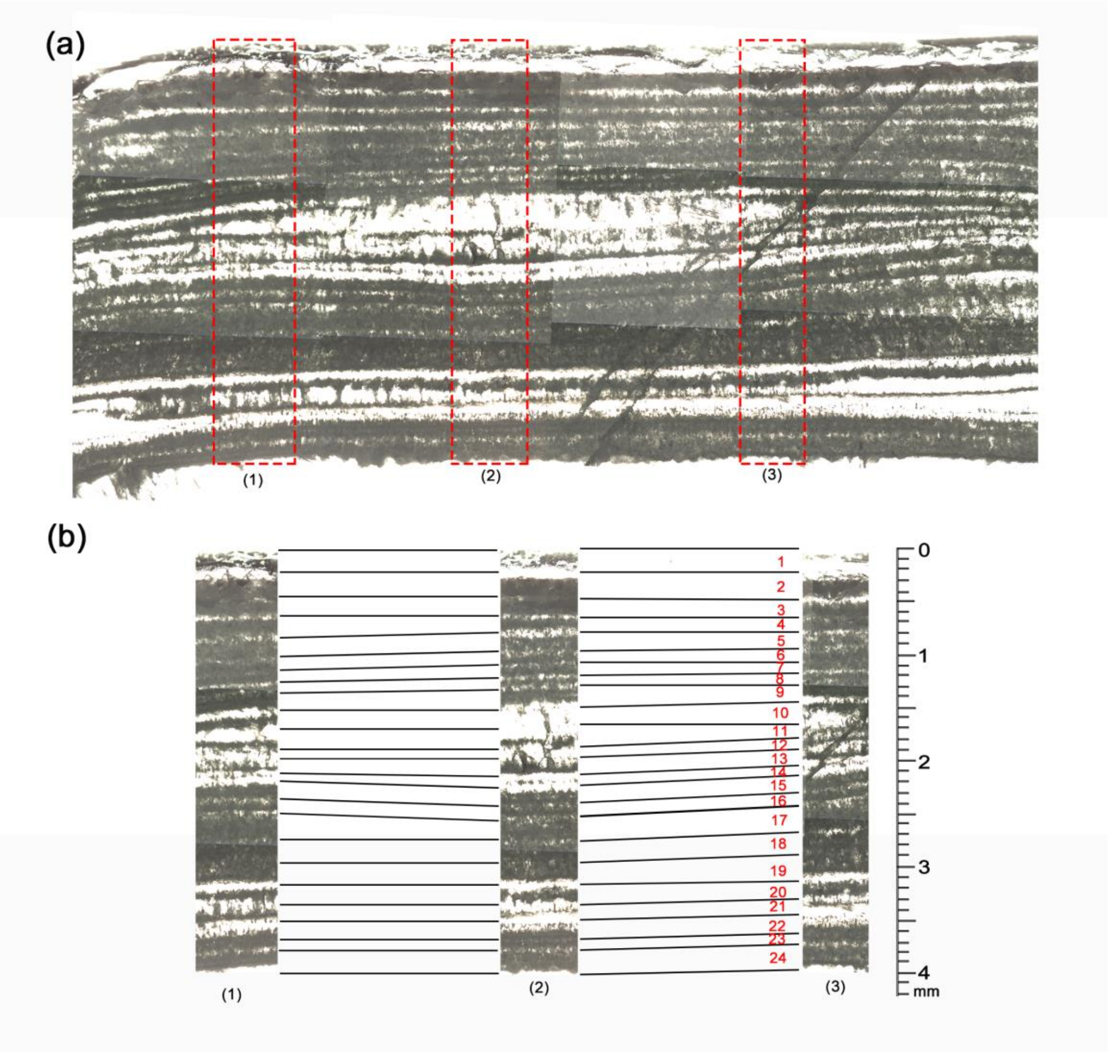

Figure S1. Microscopic image of thin section of stalagmite XMG-1 for top 4mm. (**a**) The composite microscope images. (**b**) Three columns corresponding to three red dashed-line rectangles respectively in the image (**a**). The red numbers refer to the opaque layers.

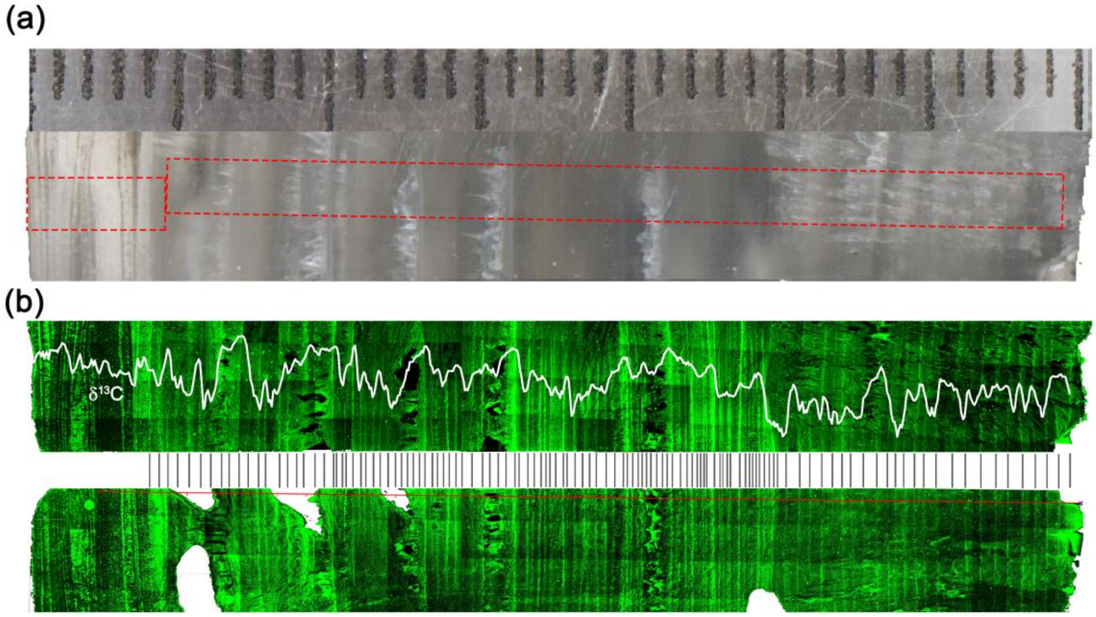

Figure S2. The scan pictures and confocal fluorescence images of XMG-1. (**a**) The image of the XMG-1 slab. Red rectangles show the micromill sampling track for stable isotope analysis. (**b**) The confocal images from two orthogonal surfaces of XMG-1 sample with the upper one corresponding to the surface in (**a**). The fluorescence layers are indicated by gray lines. The $\delta^{13}C$ analysis results are shown in white curve.

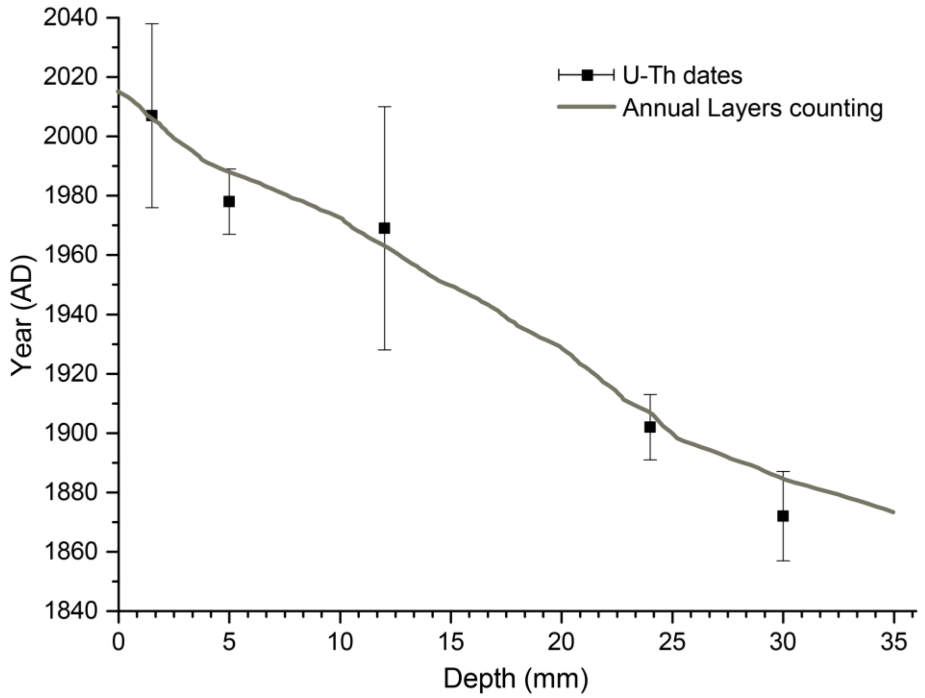

Figure S3. Age models of stalagmite XMG-1. The black solid squares with error bar indicate the five ^{230}Th dates. The gray line is the chronology constructed by annual layer counting.

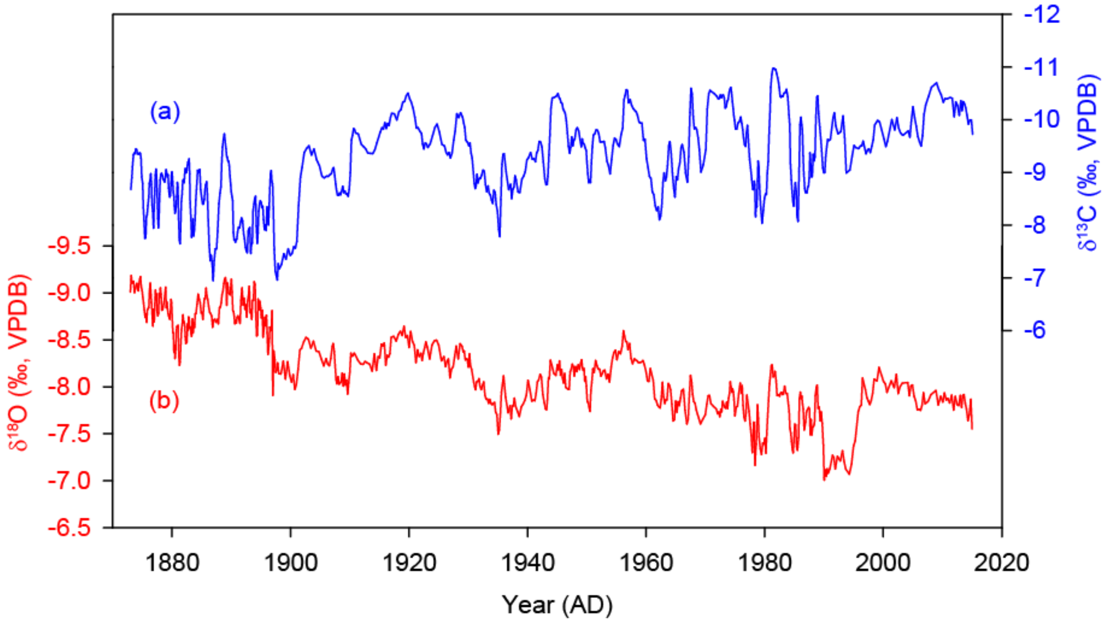

Figure S4. **The reconstructed XMG-1 stalagmite records since 1850 AD.** (**a**) $\delta^{13}C$ curve. (**b**) $\delta^{18}O$ curve.

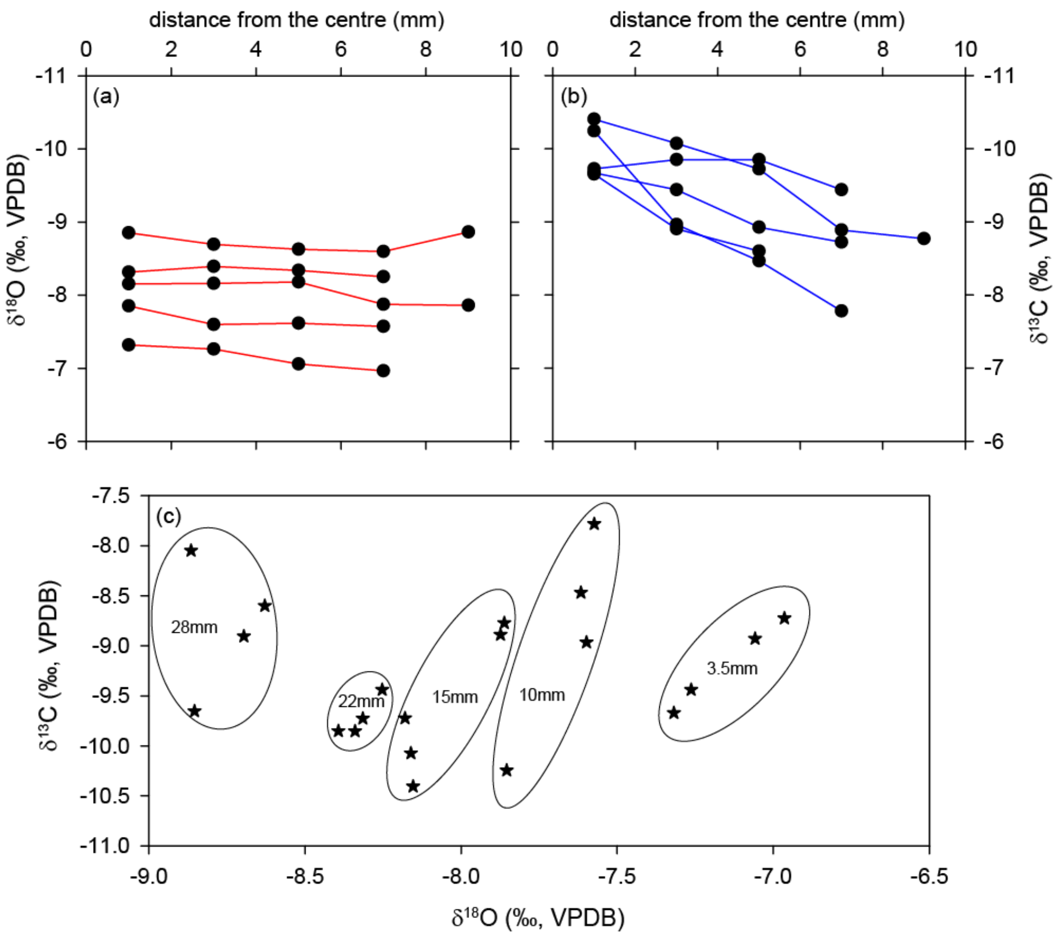

Figure S5. Hendy Test results of the sample XMG-1. (**a**) and (**b**) indicate δ^{18}O (red) and δ^{13}C (blue) values along the same growth layer at five different depths, respectively; (**c**) the co-variances between δ^{18}O and δ^{13}C at 5 different depths as labeled.

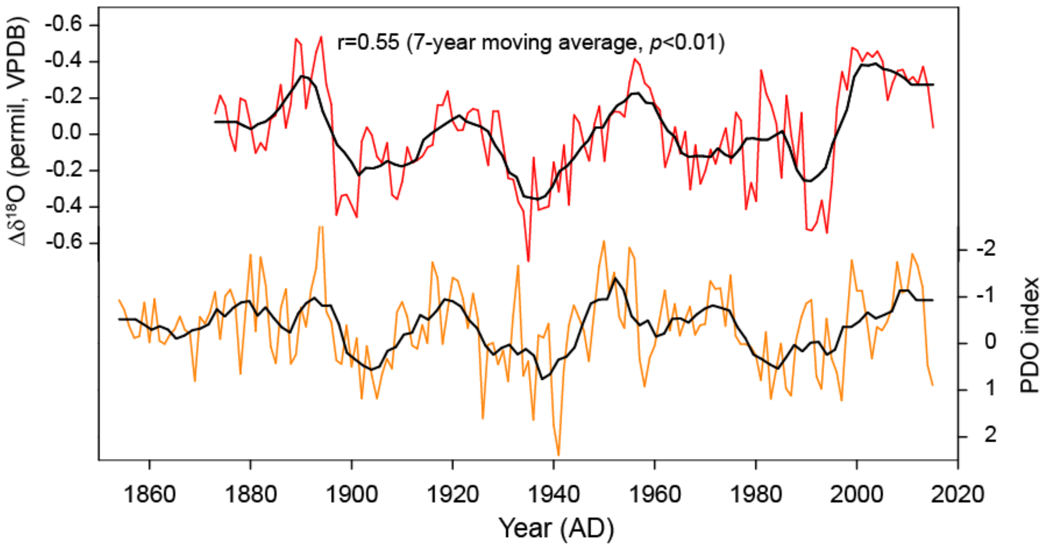

Figure S6. Comparison between the detrended Shihua $\delta^{18}O$ record and PDO index Red curve is the detrended Shihua $\delta^{18}O$ record ($\Delta\delta^{18}O$, this study). The PDO index (orange) is available at http://www.ncdc.noaa.gov/teleconnections/pdo/).The black bold curves show the 7-year moving average. The 95% confidence interval [0.47 0.61] for the Pearson correlation coefficient is derived from pairwise moving-block bootstrap resampling and calibration to preserve the serial dependence of each time series (ref. 64).

Supplementary Tables

Table S1. ^{230}Th datingresults for the XMG-1stalagmite (error is 2σ).

Depth (mm)	^{238}U (ppb)	^{232}Th (ppt)	$^{230}Th/^{232}Th$ (atomic x10^{-6})	$\delta^{234}U$* (measured)	$^{230}Th/^{238}U$ (activity)	^{230}Th age (uncorrected)	Age (AD) (corrected)	$\delta^{234}U_{Initial}$** (corrected)
1.5	750 ±4	1976 ±41	5 ±1	855.6 ±5.3	0.0008 ±0.0002	8 ±31	**2007 ±31**	856 ±5
5	611 ±3	136 ±3	51 ±14	854.1 ±6.2	0.0007 ±0.0002	37 ±11	**1978 ±11**	854 ±6
12	250 ±0.4	829 ±17	8 ±2	861.4 ±3.1	0.0017 ±0.0003	46 ±41	**1969 ±41**	862 ±3
24	183 ±0.4	66 ±3	92 ±9	855.6 ±3.9	0.0020 ±0.0002	113 ±11	**1902 ±11**	856 ±4
30	156 ±1	61 ±2	107 ±11	853.9 ±5.0	0.0025 ±0.0002	143 ±15	**1872 ±15**	854 ±5

U decay constants: λ_{238} = 1.55125x10^{-10} (ref. S2) and λ_{234} = 2.82206x10^{-6} (ref. 59). Th decay constant:λ_{230} = 9.1705x10^{-6} (ref. 59). *$\delta^{234}U$ = ([$^{234}U/^{238}U$]$_{activity}$ – 1)x1000. **$\delta^{234}U_{initial}$ was calculated based on ^{230}Th age (T), i.e. $\delta^{234}U_{initial}$ = $\delta^{234}U_{measured}$ x $e^{\lambda 234xT}$. Corrected ^{230}Th ages assume the initial $^{230}Th/^{232}Th$ atomic ratio of 4.4 ±2.2 x10^{-6}. Those are the values for a material at secular equilibrium, with the bulk earth $^{232}Th/^{238}U$ value of 3.8. The errors are arbitrarily assumed to be 50%.

Table S2. Oxygen and Carbon isotopic data of stalagmite XMG-1 from Shihua Cave. Depths are relative to the top (youngest surface) of the Shihua record and are measured along the growth axis. Ages are determined by annual layer counting. Oxygen and carbon isotope ratios are expressed in δ notation, and the permil values are derived with respect to the Vienna PeeDee Belemnite (VPDB) standard.

Depth (mm)	Year (AD)	$\delta^{18}O$ (VPDB,‰)	$\delta^{13}C$ (VPDB,‰)	Depth (mm)	Year (AD)	$\delta^{18}O$ (VPDB,‰)	$\delta^{13}C$ (VPDB,‰)
0.00	**2015.00**	-7.55	-9.72	1.80	**2004.40**	-7.91	-9.67
0.05	**2014.76**	-7.87	-9.99	1.85	**2004.14**	-8.05	-9.79
0.10	**2014.52**	-7.71	-9.99	1.95	**2003.23**	-8.04	-9.70
0.15	**2014.28**	-7.64	-9.91	2.00	**2002.83**	-8.00	-9.75
0.20	**2014.04**	-7.77	-10.08	2.05	**2002.52**	-7.93	-9.84
0.25	**2013.83**	-7.82	-10.22	2.10	**2002.22**	-8.13	-10.04
0.30	**2013.62**	-7.92	-10.30	2.15	**2001.86**	-7.99	-9.80
0.35	**2013.41**	-7.91	-10.34	2.20	**2001.37**	-8.06	-10.00
0.40	**2013.20**	-7.78	-10.17	2.30	**2000.64**	-7.94	-9.59
0.45	**2012.99**	-7.91	-10.36	2.35	**2000.34**	-8.03	-9.49
0.50	**2012.72**	-7.80	-10.08	2.40	**2000.05**	-8.06	-9.63
0.55	**2012.44**	-7.81	-10.26	2.50	**1999.30**	-8.21	-9.96
0.60	**2012.17**	-7.90	-10.34	2.55	**1998.94**	-8.06	-9.99
0.65	**2011.87**	-7.75	-10.06	2.65	**1998.47**	-8.08	-9.90
0.70	**2011.54**	-7.91	-10.41	2.70	**1998.23**	-7.93	-9.62
0.75	**2011.21**	-7.83	-10.42	2.75	**1997.99**	-7.86	-9.49
0.80	**2010.90**	-7.88	-10.41	2.80	**1997.74**	-7.81	-9.37
0.85	**2010.62**	-7.86	-10.39	2.85	**1997.50**	-7.85	-9.45
0.90	**2010.33**	-7.94	-10.40	2.90	**1997.25**	-7.88	-9.42
0.95	**2010.05**	-7.84	-10.33	2.95	**1997.00**	-7.97	-9.35
1.00	**2009.67**	-7.90	-10.47	3.05	**1996.53**	-8.09	-9.60
1.05	**2009.28**	-7.81	-10.57	3.10	**1996.29**	-7.80	-9.50
1.10	**2008.88**	-7.96	-10.70	3.15	**1996.05**	-7.79	-9.50
1.20	**2008.04**	-7.92	-10.62	3.20	**1995.79**	-7.74	-9.45
1.25	**2007.56**	-7.84	-10.44	3.30	**1995.26**	-7.42	-9.47
1.30	**2007.07**	-7.82	-10.27	3.35	**1994.99**	-7.37	-9.51
1.35	**2006.79**	-7.94	-10.10	3.40	**1994.64**	-7.18	-9.31
1.40	**2006.54**	-7.80	-9.70	3.45	**1994.29**	-7.07	-9.05
1.45	**2006.29**	-7.75	-9.50	3.55	**1993.68**	-7.13	-8.99
1.50	**2006.04**	-7.76	-9.58	3.60	**1993.41**	-7.20	-9.66
1.55	**2005.76**	-7.76	-9.68	3.65	**1993.13**	-7.32	-10.07
1.60	**2005.47**	-7.85	-9.88	3.70	**1992.75**	-7.21	-9.77
1.65	**2005.19**	-7.90	-10.04	3.75	**1992.26**	-7.26	-9.73
1.70	**2004.91**	-8.02	-10.25	3.80	**1991.90**	-7.13	-9.88
1.75	**2004.66**	-8.01	-9.99	3.85	**1991.67**	-7.25	-10.13

Continue to next page

Table S2(Cont.)

Depth (mm)	Year (AD)	$\delta^{18}O$ (VPDB,‰)	$\delta^{13}C$ (VPDB,‰)	Depth (mm)	Year (AD)	$\delta^{18}O$ (VPDB,‰)	$\delta^{13}C$ (VPDB,‰)
3.90	**1991.45**	-7.27	-10.03	5.95	**1985.24**	-7.60	-8.65
4.00	**1991.00**	-7.13	-9.63	6.00	**1985.09**	-7.60	-8.81
4.10	**1990.69**	-7.08	-9.60	6.05	**1984.94**	-7.42	-8.60
4.15	**1990.53**	-7.13	-9.62	6.10	**1984.81**	-7.30	-8.36
4.20	**1990.37**	-7.05	-9.48	6.15	**1984.67**	-7.33	-8.44
4.25	**1990.22**	-7.12	-9.30	6.20	**1984.54**	-7.42	-8.64
4.30	**1990.06**	-7.01	-9.00	6.25	**1984.41**	-7.51	-8.86
4.35	**1989.89**	-7.38	-8.99	6.30	**1984.27**	-7.56	-9.16
4.40	**1989.72**	-7.61	-9.09	6.35	**1984.14**	-7.74	-9.90
4.45	**1989.54**	-7.73	-9.40	6.40	**1984.01**	-7.80	-10.09
4.50	**1989.37**	-7.64	-9.63	6.45	**1983.80**	-7.93	-10.44
4.55	**1989.19**	-7.79	-9.87	6.50	**1983.59**	-7.93	-10.58
4.60	**1989.02**	-7.77	-10.11	6.55	**1983.38**	-8.01	-10.51
4.65	**1988.86**	-8.02	-10.45	6.60	**1983.18**	-7.92	-10.50
4.70	**1988.71**	-7.99	-10.43	6.65	**1982.98**	-7.92	-10.44
4.75	**1988.56**	-7.91	-10.05	6.70	**1982.83**	-7.94	-10.45
4.80	**1988.41**	-7.61	-9.30	6.75	**1982.68**	-7.93	-10.43
4.85	**1988.25**	-7.58	-9.22	6.80	**1982.53**	-7.90	-10.45
4.90	**1988.10**	-7.57	-9.35	6.85	**1982.39**	-7.91	-10.61
4.95	**1987.96**	-7.49	-8.93	6.90	**1982.24**	-7.92	-10.72
5.00	**1987.83**	-7.65	-8.91	6.95	**1982.09**	-7.91	-10.80
5.05	**1987.70**	-7.49	-9.10	7.00	**1981.94**	-8.06	-10.88
5.10	**1987.56**	-7.76	-9.37	7.05	**1981.77**	-8.16	-10.96
5.15	**1987.43**	-7.78	-8.89	7.10	**1981.61**	-8.12	-10.96
5.20	**1987.30**	-7.77	-8.74	7.15	**1981.44**	-8.11	-10.98
5.25	**1987.17**	-7.75	-8.72	7.20	**1981.28**	-8.23	-10.97
5.30	**1987.04**	-7.73	-8.61	7.25	**1981.11**	-8.23	-10.86
5.35	**1986.91**	-7.63	-8.64	7.30	**1980.95**	-8.10	-10.61
5.40	**1986.78**	-7.58	-8.62	7.35	**1980.80**	-8.03	-10.15
5.45	**1986.66**	-7.54	-8.70	7.40	**1980.66**	-7.86	-9.75
5.50	**1986.53**	-7.76	-8.97	7.45	**1980.51**	-7.69	-9.21
5.55	**1986.40**	-7.82	-9.32	7.50	**1980.36**	-7.50	-8.99
5.60	**1986.27**	-7.90	-9.82	7.55	**1980.21**	-7.29	-8.58
5.65	**1986.15**	-7.87	-9.92	7.60	**1980.07**	-7.45	-8.59
5.70	**1986.02**	-7.94	-10.05	7.65	**1979.88**	-7.39	-8.51
5.75	**1985.87**	-7.88	-9.91	7.70	**1979.68**	-7.40	-8.28
5.80	**1985.71**	-7.46	-9.02	7.75	**1979.47**	-7.28	-8.03
5.85	**1985.56**	-7.32	-8.07	7.80	**1979.26**	-7.34	-8.31
5.90	**1985.40**	-7.36	-8.16	7.85	**1979.05**	-7.47	-8.73

Continue to next page

Table S2(Cont.)

Depth (mm)	Year (AD)	δ^{18}O (VPDB,‰)	δ^{13}C (VPDB,‰)	Depth (mm)	Year (AD)	δ^{18}O (VPDB,‰)	δ^{13}C (VPDB,‰)
7.90	**1978.92**	-7.67	-9.11	9.90	**1972.77**	-7.73	-10.46
7.95	**1978.81**	-7.81	-9.22	9.95	**1972.60**	-7.76	-10.42
8.00	**1978.70**	-7.63	-8.84	10.00	**1972.44**	-7.68	-10.47
8.05	**1978.59**	-7.39	-8.35	10.05	**1972.27**	-7.73	-10.40
8.10	**1978.48**	-7.32	-8.42	10.10	**1972.11**	-7.71	-10.39
8.15	**1978.38**	-7.16	-8.16	10.15	**1971.83**	-7.77	-10.45
8.20	**1978.27**	-7.61	-8.76	10.20	**1971.37**	-7.79	-10.50
8.25	**1978.16**	-7.62	-8.84	10.25	**1970.95**	-7.82	-10.53
8.30	**1978.05**	-7.48	-8.68	10.30	**1970.71**	-7.91	-10.56
8.35	**1977.91**	-7.30	-8.68	10.35	**1970.47**	-7.91	-10.49
8.40	**1977.72**	-7.48	-8.87	10.40	**1970.23**	-7.81	-10.21
8.45	**1977.54**	-7.61	-9.08	10.45	**1969.98**	-7.70	-9.43
8.50	**1977.36**	-7.74	-9.25	10.50	**1969.58**	-7.65	-9.15
8.60	**1977.00**	-7.91	-9.89	10.55	**1969.18**	-7.60	-9.01
8.65	**1976.84**	-7.72	-9.76	10.60	**1968.89**	-7.67	-9.43
8.70	**1976.69**	-7.64	-9.49	10.65	**1968.68**	-7.71	-9.66
8.75	**1976.53**	-7.68	-9.53	10.70	**1968.47**	-7.76	-9.81
8.80	**1976.38**	-7.80	-9.65	10.75	**1968.26**	-7.83	-9.85
8.85	**1976.22**	-8.01	-9.81	10.80	**1968.05**	-7.84	-9.83
8.90	**1976.07**	-7.93	-9.93	10.85	**1967.86**	-7.92	-10.06
8.95	**1975.87**	-8.04	-10.05	10.90	**1967.67**	-8.06	-10.49
9.00	**1975.65**	-7.93	-9.96	10.95	**1967.49**	-8.12	-10.60
9.05	**1975.42**	-7.79	-9.94	11.00	**1967.30**	-7.92	-10.14
9.10	**1975.20**	-7.78	-9.82	11.05	**1967.11**	-7.65	-9.30
9.15	**1974.98**	-7.69	-9.82	11.10	**1966.89**	-7.61	-8.81
9.20	**1974.85**	-7.87	-9.99	11.15	**1966.59**	-7.75	-8.98
9.25	**1974.72**	-7.95	-10.08	11.20	**1966.30**	-7.97	-9.38
9.30	**1974.59**	-7.98	-10.21	11.25	**1966.01**	-7.96	-9.53
9.35	**1974.46**	-7.94	-10.35	11.30	**1965.80**	-7.88	-9.47
9.40	**1974.33**	-8.00	-10.52	11.35	**1965.60**	-7.80	-9.17
9.45	**1974.20**	-8.06	-10.62	11.40	**1965.41**	-7.84	-9.07
9.50	**1974.07**	-7.93	-10.58	11.45	**1965.21**	-7.73	-8.86
9.55	**1973.92**	-7.88	-10.52	11.50	**1965.01**	-7.85	-8.92
9.60	**1973.76**	-7.77	-10.38	11.55	**1964.79**	-7.65	-8.53
9.65	**1973.59**	-7.84	-10.35	11.60	**1964.57**	-7.64	-8.71
9.70	**1973.43**	-7.77	-10.25	11.65	**1964.36**	-7.82	-8.99
9.75	**1973.26**	-7.78	-10.22	11.70	**1964.14**	-7.97	-9.37
9.80	**1973.10**	-7.80	-10.41	11.75	**1963.93**	-8.02	-9.59
9.85	**1972.93**	-7.85	-10.47	11.80	**1963.74**	-8.04	-9.51

Continue to next page

Table S2(Cont.)

Depth (mm)	Year (AD)	$\delta^{18}O$ (VPDB,‰)	$\delta^{13}C$ (VPDB,‰)	Depth (mm)	Year (AD)	$\delta^{18}O$ (VPDB,‰)	$\delta^{13}C$ (VPDB,‰)
11.85	**1963.55**	-7.86	-9.12	13.80	**1954.35**	-8.31	-9.51
11.90	**1963.35**	-7.87	-8.92	13.85	**1954.11**	-8.21	-9.25
11.95	**1963.16**	-7.82	-8.84	13.90	**1953.85**	-8.05	-8.97
12.00	**1962.97**	-7.90	-8.99	13.95	**1953.58**	-8.14	-9.12
12.05	**1962.76**	-8.05	-8.82	14.00	**1953.32**	-8.08	-9.21
12.10	**1962.56**	-7.86	-8.36	14.05	**1953.05**	-8.18	-9.46
12.15	**1962.36**	-7.77	-8.17	14.10	**1952.82**	-8.17	-9.59
12.20	**1962.16**	-7.79	-8.10	14.15	**1952.61**	-8.14	-9.58
12.25	**1961.95**	-7.80	-8.35	14.20	**1952.39**	-8.11	-9.58
12.30	**1961.71**	-7.86	-8.27	14.25	**1952.18**	-8.19	-9.57
12.35	**1961.47**	-7.87	-8.37	14.30	**1951.96**	-8.19	-9.73
12.40	**1961.22**	-8.07	-8.58	14.35	**1951.73**	-8.22	-9.80
12.45	**1960.98**	-8.13	-8.63	14.40	**1951.49**	-8.28	-9.81
12.50	**1960.73**	-8.20	-8.89	14.45	**1951.26**	-8.15	-9.73
12.55	**1960.49**	-8.06	-8.92	14.50	**1951.03**	-8.19	-9.71
12.60	**1960.25**	-8.06	-9.09	14.55	**1950.86**	-8.05	-9.48
12.65	**1960.00**	-8.16	-9.26	14.60	**1950.71**	-7.96	-9.18
12.70	**1959.74**	-8.23	-9.63	14.65	**1950.55**	-7.74	-8.81
12.75	**1959.47**	-8.28	-9.60	14.70	**1950.39**	-7.79	-8.86
12.80	**1959.20**	-8.26	-9.94	14.75	**1950.24**	-7.83	-8.80
12.85	**1958.95**	-8.26	-9.93	14.80	**1950.08**	-7.84	-8.98
12.90	**1958.71**	-8.26	-9.96	14.85	**1949.94**	-8.01	-9.21
12.95	**1958.48**	-8.28	-10.08	14.90	**1949.80**	-8.11	-9.42
13.00	**1958.25**	-8.28	-10.15	14.95	**1949.67**	-8.06	-9.46
13.05	**1958.01**	-8.30	-10.17	15.00	**1949.54**	-8.20	-9.51
13.10	**1957.75**	-8.25	-10.21	15.05	**1949.40**	-8.18	-9.55
13.15	**1957.48**	-8.30	-10.27	15.10	**1949.27**	-8.21	-9.55
13.20	**1957.21**	-8.46	-10.38	15.15	**1949.14**	-8.29	-9.64
13.25	**1956.96**	-8.40	-10.32	15.20	**1949.00**	-8.26	-9.48
13.30	**1956.76**	-8.48	-10.56	15.25	**1948.78**	-8.18	-9.62
13.35	**1956.57**	-8.48	-10.57	15.30	**1948.55**	-8.24	-9.78
13.40	**1956.38**	-8.54	-10.45	15.35	**1948.33**	-8.16	-9.85
13.45	**1956.18**	-8.60	-10.38	15.40	**1948.10**	-8.22	-9.81
13.50	**1955.98**	-8.43	-9.84	15.45	**1947.91**	-8.12	-9.63
13.55	**1955.69**	-8.35	-9.72	15.50	**1947.73**	-8.22	-9.62
13.60	**1955.39**	-8.38	-9.49	15.55	**1947.55**	-8.24	-9.50
13.65	**1955.10**	-8.29	-9.54	15.60	**1947.38**	-8.19	-9.67
13.70	**1954.84**	-8.39	-9.62	15.65	**1947.20**	-8.16	-9.48
13.75	**1954.59**	-8.37	-9.65	15.70	**1947.03**	-7.97	-9.36

Continue to next page

Table S2(Cont.)

Depth (mm)	Year (AD)	$\delta^{18}O$ (VPDB,‰)	$\delta^{13}C$ (VPDB,‰)	Depth (mm)	Year (AD)	$\delta^{18}O$ (VPDB,‰)	$\delta^{13}C$ (VPDB,‰)
15.75	**1946.84**	-7.97	-9.49	17.70	**1937.79**	-7.88	-8.94
15.80	**1946.66**	-7.99	-9.75	17.75	**1937.60**	-7.80	-8.72
15.85	**1946.47**	-7.99	-9.98	17.80	**1937.42**	-7.80	-8.68
15.90	**1946.28**	-8.14	-10.12	17.85	**1937.23**	-7.66	-8.50
15.95	**1946.10**	-8.04	-10.17	17.90	**1937.04**	-7.80	-8.78
16.00	**1945.92**	-8.10	-10.20	17.95	**1936.69**	-7.72	-8.67
16.05	**1945.75**	-8.19	-10.30	18.00	**1936.30**	-7.89	-9.00
16.10	**1945.58**	-8.12	-10.32	18.05	**1935.95**	-8.12	-9.35
16.15	**1945.41**	-8.28	-10.40	18.10	**1935.76**	-8.03	-9.34
16.20	**1945.24**	-8.18	-10.42	18.15	**1935.58**	-7.94	-9.15
16.25	**1945.07**	-8.24	-10.50	18.20	**1935.39**	-7.71	-8.49
16.30	**1944.79**	-8.12	-10.44	18.25	**1935.20**	-7.54	-7.78
16.35	**1944.44**	-8.14	-10.42	18.30	**1935.02**	-7.49	-7.92
16.40	**1944.09**	-8.24	-10.41	18.35	**1934.82**	-7.70	-8.27
16.45	**1943.87**	-8.28	-10.22	18.40	**1934.62**	-7.73	-8.39
16.50	**1943.70**	-8.09	-9.72	18.45	**1934.42**	-7.84	-8.62
16.55	**1943.53**	-8.10	-9.33	18.50	**1934.22**	-7.82	-8.66
16.60	**1943.36**	-7.86	-8.93	18.55	**1934.02**	-7.81	-8.41
16.65	**1943.19**	-7.76	-8.77	18.60	**1933.82**	-7.82	-8.48
16.70	**1943.02**	-7.76	-8.76	18.65	**1933.62**	-7.87	-8.57
16.75	**1942.79**	-7.83	-8.88	18.70	**1933.42**	-7.82	-8.60
16.80	**1942.56**	-7.92	-9.19	18.75	**1933.22**	-7.87	-8.74
16.85	**1942.33**	-8.10	-9.48	18.80	**1933.02**	-7.87	-8.63
16.90	**1942.09**	-8.13	-9.57	18.85	**1932.81**	-7.91	-8.77
16.95	**1941.87**	-8.07	-9.59	18.90	**1932.59**	-8.05	-9.02
17.00	**1941.65**	-8.05	-9.42	18.95	**1932.38**	-8.02	-9.04
17.05	**1941.44**	-7.93	-9.37	19.00	**1932.16**	-7.97	-8.95
17.10	**1941.22**	-7.85	-9.20	19.05	**1931.96**	-8.01	-8.88
17.15	**1941.01**	-7.86	-9.26	19.10	**1931.81**	-8.09	-8.89
17.20	**1940.70**	-7.85	-9.25	19.15	**1931.67**	-8.06	-8.87
17.25	**1940.38**	-7.96	-9.38	19.20	**1931.52**	-8.06	-8.80
17.30	**1940.07**	-8.07	-9.28	19.25	**1931.37**	-8.05	-8.97
17.35	**1939.78**	-7.91	-9.24	19.30	**1931.22**	-7.97	-8.75
17.40	**1939.50**	-7.86	-9.02	19.35	**1931.08**	-8.00	-8.69
17.45	**1939.22**	-7.82	-8.93	19.40	**1930.91**	-8.05	-8.79
17.50	**1938.93**	-7.79	-8.87	19.50	**1930.55**	-8.19	-9.27
17.55	**1938.61**	-7.68	-8.62	19.55	**1930.37**	-8.22	-9.37
17.60	**1938.28**	-7.74	-8.63	19.60	**1930.19**	-8.35	-9.56
17.65	**1937.97**	-7.80	-8.86	19.65	**1930.00**	-8.21	-9.50

Continue to next page

Table S2(Cont.)

Depth (mm)	Year (AD)	$\delta^{18}O$ (VPDB,‰)	$\delta^{13}C$ (VPDB,‰)	Depth (mm)	Year (AD)	$\delta^{18}O$ (VPDB,‰)	$\delta^{13}C$ (VPDB,‰)
19.70	**1929.83**	-8.29	-9.56	21.65	**1918.86**	-8.55	-10.33
19.75	**1929.66**	-8.30	-9.59	21.70	**1918.53**	-8.59	-10.19
19.80	**1929.49**	-8.29	-9.61	21.75	**1918.20**	-8.56	-10.15
19.85	**1929.32**	-8.38	-9.85	21.80	**1917.83**	-8.49	-9.99
19.90	**1929.14**	-8.37	-9.93	21.85	**1917.40**	-8.54	-10.11
19.95	**1928.95**	-8.43	-10.05	21.90	**1916.99**	-8.53	-10.12
20.00	**1928.62**	-8.46	-10.13	21.95	**1916.76**	-8.35	-10.04
20.05	**1928.29**	-8.30	-10.04	22.00	**1916.54**	-8.32	-9.89
20.10	**1927.97**	-8.44	-10.11	22.05	**1916.31**	-8.34	-9.93
20.15	**1927.70**	-8.32	-9.78	22.10	**1916.09**	-8.23	-9.83
20.20	**1927.43**	-8.21	-9.61	22.15	**1915.84**	-8.47	-9.94
20.25	**1927.17**	-8.19	-9.37	22.20	**1915.59**	-8.43	-9.82
20.30	**1926.91**	-8.10	-9.27	22.25	**1915.33**	-8.46	-9.85
20.35	**1926.66**	-8.30	-9.38	22.30	**1915.08**	-8.31	-9.77
20.40	**1926.42**	-8.27	-9.33	22.35	**1914.78**	-8.32	-9.67
20.45	**1926.18**	-8.22	-9.38	22.40	**1914.47**	-8.17	-9.56
20.50	**1925.88**	-8.32	-9.50	22.45	**1914.16**	-8.35	-9.43
20.55	**1925.45**	-8.28	-9.53	22.50	**1913.80**	-8.17	-9.35
20.60	**1925.02**	-8.44	-9.77	22.55	**1913.37**	-8.24	-9.36
20.65	**1924.64**	-8.50	-9.92	22.60	**1912.96**	-8.25	-9.37
20.70	**1924.27**	-8.49	-9.85	22.65	**1912.61**	-8.27	-9.44
20.75	**1923.88**	-8.45	-9.70	22.70	**1912.26**	-8.24	-9.49
20.80	**1923.48**	-8.30	-9.54	22.75	**1911.87**	-8.24	-9.53
20.85	**1923.09**	-8.42	-9.47	22.80	**1911.34**	-8.29	-9.73
20.90	**1922.84**	-8.48	-9.55	22.85	**1910.93**	-8.34	-9.75
20.95	**1922.63**	-8.42	-9.56	22.90	**1910.75**	-8.36	-9.81
21.00	**1922.42**	-8.36	-9.44	22.95	**1910.56**	-8.33	-9.83
21.05	**1922.21**	-8.28	-9.54	23.00	**1910.37**	-8.33	-9.70
21.10	**1922.00**	-8.36	-9.74	23.05	**1910.19**	-8.36	-9.61
21.15	**1921.72**	-8.32	-9.78	23.10	**1910.00**	-8.15	-8.99
21.20	**1921.44**	-8.39	-9.85	23.15	**1909.82**	-8.04	-8.68
21.25	**1921.16**	-8.26	-9.84	23.20	**1909.64**	-7.92	-8.55
21.30	**1920.88**	-8.44	-10.04	23.25	**1909.46**	-8.07	-8.59
21.35	**1920.58**	-8.48	-10.22	23.30	**1909.28**	-8.02	-8.59
21.40	**1920.29**	-8.59	-10.33	23.35	**1909.10**	-8.14	-8.65
21.45	**1920.00**	-8.42	-10.40	23.40	**1908.92**	-8.01	-8.61
21.50	**1919.72**	-8.55	-10.50	23.45	**1908.74**	-8.15	-8.74
21.55	**1919.44**	-8.52	-10.45	23.50	**1908.56**	-8.05	-8.64
21.60	**1919.16**	-8.65	-10.35	23.55	**1908.38**	-8.04	-8.57

Continue to next page

Table S2(Cont.)

Depth (mm)	Year (AD)	$\delta^{18}O$ (VPDB,‰)	$\delta^{13}C$ (VPDB,‰)	Depth (mm)	Year (AD)	$\delta^{18}O$ (VPDB,‰)	$\delta^{13}C$ (VPDB,‰)
23.60	**1908.20**	-8.03	-8.61	25.55	**1897.02**	-7.91	-8.31
23.65	**1908.01**	-8.11	-8.56	25.60	**1896.90**	-8.81	-8.78
23.70	**1907.85**	-8.03	-8.57	25.65	**1896.78**	-8.61	-8.82
23.75	**1907.69**	-8.08	-8.65	25.70	**1896.66**	-8.60	-9.04
23.80	**1907.53**	-8.22	-8.78	25.75	**1896.55**	-8.64	-8.96
23.85	**1907.37**	-8.37	-8.94	25.80	**1896.43**	-8.52	-8.70
23.90	**1907.21**	-8.46	-9.09	25.85	**1896.31**	-8.41	-8.34
23.95	**1907.05**	-8.43	-9.07	25.90	**1896.19**	-8.34	-8.01
24.00	**1906.82**	-8.30	-8.97	25.95	**1896.08**	-8.58	-8.15
24.05	**1906.55**	-8.23	-8.96	26.00	**1895.95**	-8.76	-8.35
24.10	**1906.29**	-8.24	-8.92	26.05	**1895.81**	-8.65	-7.90
24.15	**1906.02**	-8.30	-8.93	26.10	**1895.67**	-8.74	-8.01
24.20	**1905.63**	-8.22	-8.90	26.15	**1895.53**	-8.62	-7.92
24.25	**1905.23**	-8.30	-8.93	26.20	**1895.39**	-8.53	-8.10
24.30	**1904.80**	-8.38	-9.13	26.25	**1895.25**	-8.82	-8.36
24.35	**1904.34**	-8.38	-9.24	26.30	**1895.11**	-8.73	-8.33
24.40	**1903.89**	-8.49	-9.44	26.35	**1894.97**	-8.84	-8.43
24.45	**1903.46**	-8.41	-9.33	26.40	**1894.83**	-8.91	-8.46
24.50	**1903.02**	-8.51	-9.51	26.45	**1894.70**	-8.82	-8.40
24.55	**1902.62**	-8.53	-9.46	26.50	**1894.57**	-8.93	-8.26
24.60	**1902.22**	-8.48	-9.38	26.55	**1894.43**	-8.55	-7.85
24.65	**1901.87**	-8.41	-9.09	26.60	**1894.30**	-8.54	-7.65
24.70	**1901.58**	-8.39	-8.91	26.65	**1894.16**	-8.79	-7.98
24.75	**1901.29**	-8.19	-8.38	26.70	**1894.03**	-9.09	-8.55
24.80	**1900.99**	-8.02	-7.69	26.75	**1893.89**	-9.12	-8.50
24.85	**1900.73**	-7.97	-7.56	26.80	**1893.76**	-8.92	-8.41
24.90	**1900.46**	-8.13	-7.59	26.85	**1893.62**	-8.92	-8.28
24.95	**1900.19**	-8.05	-7.47	26.90	**1893.48**	-8.72	-7.66
25.00	**1899.90**	-8.12	-7.43	26.95	**1893.35**	-8.82	-7.64
25.05	**1899.53**	-8.27	-7.55	27.00	**1893.21**	-8.79	-7.46
25.10	**1899.16**	-8.08	-7.35	27.05	**1893.07**	-9.07	-7.96
25.15	**1898.78**	-8.28	-7.46	27.10	**1892.93**	-8.98	-7.94
25.20	**1898.41**	-8.14	-7.25	27.15	**1892.78**	-8.91	-7.89
25.25	**1898.03**	-8.14	-7.17	27.20	**1892.62**	-8.74	-7.48
25.30	**1897.85**	-8.26	-7.27	27.25	**1892.47**	-8.87	-7.51
25.35	**1897.68**	-8.18	-6.96	27.30	**1892.32**	-8.83	-7.58
25.40	**1897.52**	-8.15	-7.05	27.35	**1892.17**	-8.95	-7.74
25.45	**1897.35**	-8.29	-7.13	27.40	**1892.02**	-8.87	-7.83
25.50	**1897.19**	-8.38	-7.54	27.45	**1891.84**	-9.06	-8.08

Continue to next page

Table S2(Cont.)

Depth (mm)	Year (AD)	$\delta^{18}O$ (VPDB,‰)	$\delta^{13}C$ (VPDB,‰)	Depth (mm)	Year (AD)	$\delta^{18}O$ (VPDB,‰)	$\delta^{13}C$ (VPDB,‰)
27.50	**1891.67**	-8.92	-8.14	29.45	**1886.04**	-8.93	-8.09
27.55	**1891.49**	-8.68	-8.06	29.50	**1885.90**	-8.97	-8.58
27.60	**1891.32**	-8.72	-7.95	29.55	**1885.77**	-9.05	-8.82
27.65	**1891.14**	-8.76	-7.96	29.60	**1885.63**	-8.94	-8.76
27.70	**1890.98**	-8.74	-7.86	29.65	**1885.50**	-8.94	-8.68
27.75	**1890.86**	-8.68	-7.73	29.70	**1885.37**	-8.86	-8.49
27.80	**1890.74**	-8.71	-7.73	29.75	**1885.23**	-8.72	-8.40
27.85	**1890.63**	-8.67	-7.68	29.80	**1885.10**	-8.79	-8.40
27.90	**1890.51**	-8.76	-7.72	29.85	**1884.95**	-8.78	-8.50
27.95	**1890.39**	-8.80	-7.72	29.90	**1884.78**	-8.90	-8.67
28.00	**1890.28**	-8.81	-7.87	29.95	**1884.60**	-8.93	-9.06
28.05	**1890.16**	-9.01	-8.47	30.00	**1884.43**	-8.99	-9.00
28.10	**1890.04**	-9.15	-8.54	30.05	**1884.25**	-8.88	-9.04
28.15	**1889.93**	-9.04	-8.64	30.10	**1884.08**	-8.78	-8.68
28.20	**1889.82**	-8.98	-8.77	30.15	**1883.93**	-8.78	-8.48
28.25	**1889.70**	-8.99	-8.94	30.20	**1883.82**	-8.63	-8.11
28.30	**1889.59**	-9.00	-9.13	30.25	**1883.70**	-8.68	-7.90
28.35	**1889.48**	-9.06	-9.21	30.30	**1883.58**	-8.57	-7.80
28.40	**1889.37**	-9.11	-9.21	30.35	**1883.47**	-8.72	-8.11
28.45	**1889.25**	-8.87	-9.29	30.40	**1883.35**	-8.54	-7.87
28.50	**1889.14**	-9.10	-9.30	30.45	**1883.23**	-8.57	-7.77
28.55	**1889.03**	-9.16	-9.45	30.50	**1883.12**	-8.64	-8.14
28.60	**1888.90**	-9.14	-9.60	30.55	**1883.00**	-8.62	-8.41
28.65	**1888.75**	-9.10	-9.73	30.60	**1882.91**	-8.73	-8.97
28.70	**1888.61**	-9.01	-9.64	30.65	**1882.81**	-8.75	-9.26
28.75	**1888.46**	-8.98	-9.48	30.70	**1882.72**	-8.60	-9.16
28.80	**1888.32**	-8.90	-9.21	30.75	**1882.63**	-8.67	-9.20
28.85	**1888.18**	-8.85	-8.73	30.80	**1882.54**	-8.47	-9.14
28.90	**1888.03**	-8.85	-8.61	30.85	**1882.45**	-8.48	-9.05
28.95	**1887.84**	-8.67	-8.41	30.90	**1882.35**	-8.46	-9.00
29.00	**1887.64**	-8.72	-7.89	30.95	**1882.26**	-8.53	-8.99
29.05	**1887.44**	-8.69	-7.55	31.00	**1882.17**	-8.64	-9.07
29.10	**1887.24**	-8.72	-7.55	31.05	**1882.08**	-8.69	-8.99
29.15	**1887.04**	-8.71	-7.33	31.10	**1881.98**	-8.68	-8.81
29.20	**1886.87**	-8.63	-6.95	31.15	**1881.86**	-8.77	-8.87
29.25	**1886.70**	-8.75	-7.26	31.20	**1881.75**	-8.67	-8.84
29.30	**1886.54**	-8.78	-7.43	31.25	**1881.63**	-8.61	-8.58
29.35	**1886.37**	-8.79	-7.32	31.30	**1881.51**	-8.45	-8.45
29.40	**1886.21**	-8.86	-7.62	31.35	**1881.40**	-8.27	-7.93

Continue to next page

Table S2(Cont.)

Depth (mm)	Year (AD)	$\delta^{18}O$ (VPDB,‰)	$\delta^{13}C$ (VPDB,‰)	Depth (mm)	Year (AD)	$\delta^{18}O$ (VPDB,‰)	$\delta^{13}C$ (VPDB,‰)
31.40	**1881.28**	-8.23	-7.65	33.30	**1877.23**	-9.03	-9.06
31.45	**1881.16**	-8.33	-7.72	33.35	**1877.13**	-8.87	-8.94
31.50	**1881.04**	-8.66	-8.13	33.40	**1877.03**	-8.69	-8.66
31.55	**1880.94**	-8.64	-8.71	33.45	**1876.92**	-8.83	-8.28
31.60	**1880.84**	-8.64	-8.81	33.50	**1876.80**	-8.65	-7.94
31.65	**1880.74**	-8.58	-8.54	33.55	**1876.69**	-8.76	-8.26
31.70	**1880.64**	-8.40	-8.39	33.60	**1876.58**	-8.90	-8.38
31.75	**1880.54**	-8.30	-8.27	33.65	**1876.46**	-8.99	-8.77
31.80	**1880.45**	-8.44	-8.22	33.70	**1876.35**	-9.10	-8.97
31.85	**1880.35**	-8.37	-8.37	33.75	**1876.23**	-9.02	-8.85
31.90	**1880.25**	-8.58	-8.55	33.80	**1876.12**	-8.84	-8.79
31.95	**1880.15**	-8.58	-8.62	33.85	**1876.01**	-8.83	-8.54
32.05	**1879.95**	-8.90	-9.01	33.90	**1875.88**	-8.82	-8.37
32.10	**1879.85**	-8.93	-9.00	33.95	**1875.75**	-8.69	-8.20
32.15	**1879.76**	-8.80	-8.79	34.00	**1875.62**	-8.78	-8.16
32.20	**1879.66**	-8.72	-8.56	34.05	**1875.49**	-8.74	-7.76
32.25	**1879.56**	-8.74	-8.53	34.10	**1875.36**	-8.82	-7.75
32.30	**1879.46**	-8.76	-8.59	34.15	**1875.23**	-8.90	-8.08
32.35	**1879.36**	-8.78	-8.70	34.20	**1875.10**	-8.95	-8.33
32.40	**1879.26**	-8.85	-8.96	34.25	**1874.97**	-9.03	-8.79
32.45	**1879.16**	-8.91	-8.91	34.30	**1874.84**	-9.07	-8.92
32.50	**1879.06**	-8.84	-8.96	34.35	**1874.72**	-9.17	-9.27
32.55	**1878.96**	-9.06	-9.05	34.40	**1874.60**	-9.14	-9.36
32.65	**1878.70**	-8.91	-8.82	34.45	**1874.47**	-9.09	-9.37
32.75	**1878.45**	-8.82	-8.87	34.50	**1874.35**	-9.02	-9.34
32.80	**1878.32**	-8.82	-8.94	34.55	**1874.22**	-9.05	-9.36
32.85	**1878.19**	-8.98	-8.99	34.60	**1874.10**	-9.10	-9.35
32.90	**1878.06**	-9.05	-8.96	34.65	**1873.97**	-9.08	-9.44
32.95	**1877.95**	-8.95	-8.86	34.70	**1873.83**	-9.04	-9.45
33.00	**1877.85**	-9.00	-8.59	34.75	**1873.70**	-9.00	-9.36
33.05	**1877.74**	-8.78	-8.22	34.80	**1873.56**	-9.13	-9.37
33.10	**1877.64**	-8.76	-7.95	34.85	**1873.42**	-9.12	-9.33
33.15	**1877.54**	-8.79	-8.13	34.90	**1873.28**	-9.10	-9.11
33.20	**1877.44**	-8.99	-8.64	34.95	**1873.15**	-9.19	-8.90
33.25	**1877.34**	-8.93	-8.92	35.00	**1873.01**	-9.01	-8.67

References

S1. Scholz, D. & Hoffmann, D. L. StalAge–An algorithm designed for construction of speleothem age models. *Quat. Int.* **6,** 369-382 (2011).

S2. Jaffey, A.H., Flynn, K.F., Glendenin, L.E., Bentley, W.C.& Essling, A.M. Precision measurement of half-lives and specific activities of ^{235}U and ^{238}U. *Phys. Rev.* **C4,** 1889-1906 (1971).
